# Poor Adherence to the Integrated Community Case Management of Newborn and Child Illness Protocol in Rural Ethiopia

**DOI:** 10.4269/ajtmh.21-1018

**Published:** 2022-10-31

**Authors:** Solomon Abtew, Mariamawit Negatou, Tamiru Wondie, Yenealem Tadesse, Wondwossen A. Alemayehu, Dawit A. Tsegaye, Mitswat Mulaw, Dagne Muluneh, Deborah Collison, Eden Ahmed Mdluli, Legese A. Mekuria

**Affiliations:** ^1^ Project HOPE, Assosa, Ethiopia;; ^2^ Project HOPE, Addis Ababa, Ethiopia;; ^3^ Project HOPE, Washington, District of Columbia

## Abstract

Ethiopia has adopted the Integrated Community Case Management of Newborn and Child Illness (iCMNCI) strategy to expand access to neonatal and child health services. This study assessed compliance with the iCMNCI case management protocol at the primary care settings. A descriptive cross-sectional assessment was conducted in eight districts of Benishangul-Gumuz Region from April to December 2019, and 1,217 sick children aged 2 to 59 months and 43 sick young infants aged 0 to 2 months who sought clinical consultation at the 236 health posts were selected purposively. Trained supervisors reviewed the medical records of two most recent cases from each illness category to quantify the extent to which health workers correctly assessed, classified, treated, and followed up cases per the iCMNCI guidelines. A total of 32,981 children sought clinical consultation of whom 31,830 (96.5%) were aged 2 to 59 months, and 1,151 (3.5%) were young infants aged 0 to 2 months. Of the 1,217 selected children, 426 (35%) had pneumonia, 287 (23.6%) malaria, 501 (41.2%) diarrhea, and 3 (0.2%) had malnutrition. Nearly two-thirds 306 (72%) of pneumonia cases were correctly classified as having had the disease and 297 (70%) were correctly treated for pneumonia; 213 (74%) were correctly classified as having had malaria and 210 (73%) were correctly treated for malaria; and 393 (78%) were correctly classified as having had diarrhea and 297 (59%) were correctly treated for diarrhea. Generally, the current practices of child illness assessment, classification, and treatment have deviated from iCMNCI guidelines. Future interventions should support frontline health workers to comply strictly with case management protocols through training, mentorship, and supervision.

## INTRODUCTION

Globally, substantial progress has been made in reducing child deaths since 1990. The total number of under-5 deaths worldwide has declined from 12.6 million in 1990 to 5.3 million in 2018.^1^ The global under-5 mortality rate also dropped from 93 deaths per 1,000 live births in 1990 to 39 deaths in 2018.^1^ However, disparities exist across regions and countries with sub-Saharan Africa remaining the region with the highest under-5 mortality worldwide.^1^

Ethiopia has made remarkable progress in achieving the Millennium Development Goal 4 of reducing child mortality by two-thirds between 1990 and 2015. However, the current under-5 and neonatal mortality rates remain unacceptably high.^2^ According to the mini-Ethiopian Demographic Health Survey (EDHS) 2019 report, under-5 deaths decreased from 123 to 59 deaths per 1,000 live births, infant mortality reduced from 77 to 47 deaths per 1,000 live births, and neonatal mortality from 39 to 33 deaths per 1,000 live births compared with the 2005 EDHS report.^3^ The leading causes of death for newborns include preterm birth complications, birth asphyxia, and sepsis.^2^^,^^4^^,^^5^ Pneumonia, diarrhea, and malaria are the foremost causes of under-5 mortality, with malnutrition as an important underlying cause.^4^

According to the EDHS 2016 report, Benishangul Gumuz Region has the second highest under-5 child mortality in Ethiopia having had 98 deaths per 1,000 live births and with neonatal and infant mortality rates of 35 and 62 per 1,000 live births, respectively.^6^ The Sustainable Development Goal of reducing under-5 mortality to 25 per 1,000 live births by 2030 is still far from being achieved in this region.^7^

Most under-5 deaths can be prevented by simple, cost-effective, and affordable interventions such as oral rehydration salt (ORS) and zinc for diarrhea, antibiotic treatment of pneumonia, and artemisinin-based combination therapy for malaria.^8^^–^^13^ However, poor and inequitable access to primary health care,^14^^,^^15^ and the low-quality care at home and at the facility level^15^^,^^16^ are affecting survival of under-5 children greatly.

In response to the high child mortalities in Ethiopia, the Integrated Community Management of Newborn and Childhood Illness (iCMNCI) previously known as Integrated Community Case Management strategy was introduced to expand access to basic child health services, particularly in primary care settings with limited access to skilled health workers and essential medicines. Experiences from low- and middle-income countries (LMICs) suggest that iCMNCI helps frontline health workers to deliver quality health services to sick children in community settings where access to basic healthcare is limited.^17^^,^^18^

Ethiopia has been implementing the iCMNCI strategy since 2010 to treat uncomplicated pneumonia, diarrhea, malaria, malnutrition, and measles by health extension workers (HEWs).^19^ In collaboration with local and global development partners, the Ethiopian government has also integrated the iCMNCI guidelines into the national health policy to maintain service quality in the primary care settings. Despite the efforts, low utilization and poor consistency of care persist in rural settings.^20^^,^^21^ Assessment of health services for under-5 children suggest that the quality of care and compliance with the guidelines vary greatly. Key components of the iCMNCI strategy including training, follow-up, clinical mentorship, supervision, and provision of commodities^22^^–^^24^ affect the compliance of HEWs with guideline recommendations.

The iCMNCI process follows five steps: assess the young infant or child, classify the illness, identify treatment and treat the young infant or the child, counsel the mother, and, finally, give follow-up care for the sick young infant or child.^25^ To improve the quality of iCMNCI services in rural Ethiopia, performance review and clinical mentorship meetings (PRCMM) have been executed successfully.^26^ But, the consistency of child illness assessment, disease classification, treatment, and follow-up by rural HEWs were generally low.^27^ A study conducted in LMICs has also reported low iCMNCI treatment coverage. In addition, the quality of iCMNCI services and the caseload after iCMNCI intervention is not known.^28^ To the best of our understanding, the quality of child illness assessment, disease classification, treatment, and follow-up of newborns and children aged 0 to 59 months have not been assessed in Benishangul-Gumuz region. This study was conducted to assess the compliance of child illness assessment, classification, treatment, and follow-up practices with national iCMNCI guideline recommendations at the health post level.

## METHODS AND MATERIALS

### Study setting.

A descriptive cross-sectional assessment was conducted from April to December 2019 in eight districts of Benishangul Gumuz Region, North-West Ethiopia. The region has an estimated population of 1,141,277^29^ and is located 680 km away from the capital, Addis Ababa. It has three administrative zones, one special district, and 210 districts and three town administrations. There are two general hospitals, four primary hospitals, 58 health centers, and 408 health posts. The USAID-funded Transform Health in Developing Regions (USAID T-HDR) project supports 10 districts, one general hospital, three primary hospitals, 29 health centers, and 248 health posts in the region. At the time of this study, all the health posts were providing iCMNCI services to under-5 children.

### Study population.

Study participants were all under-5 children who received care at the health posts of Benishangul-Gumuz Region between April and December 2019.

### Sampling technique and sampling procedure.

**Figure 1.**
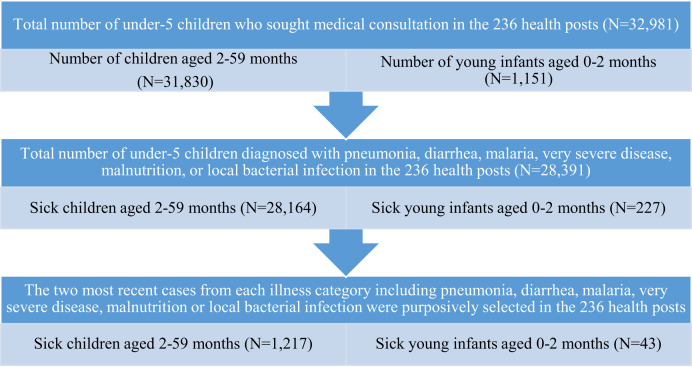
Purposive sampling procedure, Benishangul Gumuz Region, Ethiopia, 2019.

Purposive sampling technique was applied by taking the most recently treated under-5 children for the common childhood illnesses like pneumonia, diarrhea, malnutrition, and malaria. A total of 1,217 sick children aged 2 to 59 months and 43 sick young infants (SYI) aged 0 to 2 months were sampled. First, all under-5 children who sought medical consultation for common childhood illnesses including pneumonia, diarrhea, malnutrition, and malaria were identified. Next, the two most recent cases of each illness category were selected purposively, and a total of 1,217 sick children aged 2 to 59 months and 43 SYIs aged 0 to 2 months were included in the study (Figure 1).

### Inclusion and exclusion criteria.

**Inclusion criteria.** All SYI and children who received clinical care in the 236 health posts from April to December 2019 were included in the study.
**Exclusion criteria.** SYI and children whose records were incomplete or not easily accessible were excluded. In addition, children who presented with tonsilitis, burn, or skin infection that were recorded as “other cases” were excluded because these cases are not included in the iCMNCI guideline.

### Study procedure.

Before the study, HEWs had attended the iCMNCI training and had received a chart booklet and two registration books to record SYI aged 0 to 2 months and sick children aged 2 to 59 months. The iCMNCI targets six childhood illnesses: pneumonia, diarrhea, malaria, malnutrition, very severe disease, and local bacterial infection. Therefore, patients’ records were reviewed to select the two most recent cases of each illness category and to assess whether child illness assessment, classification, treatment, and follow-up were in accordance with the iCMNCI guidelines.

### Data collection.

Secondary data on the iCMNCI register were reviewed from November to December 2019. Individual case data were collected during the PRCMM when all HEWs from catchment Kebeles brought together with patient registration logbooks and the chart booklet (iCMNCI guideline) for performance review. The PRCMM is 2 days review meeting in which iCMNCI registers and logbooks were reviewed and examined by trained facilitators on the first day followed by clinical mentorship.^27^ Trained health workers facilitated the PRCMM for 15 to 20 HEWs in a central town hall or at a primary healthcare setting. Data collectors and peer-HEWs reviewed the registers for consistency, completeness, and numbers of observed versus expected caseloads. One-to-one feedback and experience sharing were made among HEWs as they reviewed each other’s registers in a good spirit.^27^

Data collection forms including data abstraction, tally sheet, and follow-up Form “C” were adopted from the PRCMM guide.^30^ Data abstraction template/tally sheet was used to collect all case-based records on the iCMNCI registration book and follow-up Form “C” was used to collect the two most recent cases that had been recorded just before the conduct of PRCMM.

Data were abstracted from patient records to assess the caseload of childhood illnesses at the health posts. Two trained data collectors and 16 to 20 HEWs (two HEWs from each health post) sat together to review if the two most recent cases of each illness category had been assessed, classified, treated, and followed up in accordance with the iCMNCI guidelines. This was conducted by trained and experienced data collectors from regional health bureau, zonal health department, district health office, HEW supervisors, and project staff. More than 10 clinical officers who were trained on iCMNCI abstracted the data, and cases were reviewed together with HEWs. The whole process of data collection was supervised and coordinated by two program officers and one supervisor from the regional health bureau.

### Operational definition of key terms.

**Case/most recent case.** *Case* refers to under-5 child classified as having pneumonia, malaria, diarrhea, malnutrition, very severe disease, and/or local bacterial infection and *most recent case* means the case that had been assessed by the community health workers (such as HEWs) most recently before the PRCMM review. Recent indicates time. If the HEWs assessed 10 malaria cases in a month at a health post, only the two most recent cases were included in the study per the iCMNCI guideline.
**Correct assessment.** Completeness of the assessment to the sick child and young infant by HEWs according to the iCMNCI clinical guideline.
**Classification.** The probable illness assigned to the sick child and SYI after the assessment by HEWs.
**Correct classification.** All classifications of sick children and SYIs made by HEWs that were matched to the assessment result per the iCMNCI clinical guideline.
**Correct treatment.** All treatments of sick children and SYIs made by HEWs that were matched to the classification results per the iCMNCI clinical guideline.
**Correct follow-up status.** All follow-up dates made by HEWs were matched to the classification per the iCMNCI guideline.
**Compliance with classification guidelines.** How many cases are correctly classified according to the iCMNCI guideline recommendation?
**Compliance with treatment guidelines.** How many cases are correctly treated according to the iCMNCI guideline recommendation? ICMNCI guideline classification and case management includes a combination of signs and symptoms that lead to a child’s classification within one or more symptom groups rather than a diagnosis. The classification of an illness is based on a color-coded triage system: pink indicates urgent hospital referral or admission, yellow indicates initiation of specific outpatient treatment, and green indicates supportive home care.
**Compliance with follow-up guidelines.** How many cases have follow-up dates correctly stated on patient records per the iCMNCI guideline recommendation?
**Child/Children.** Children’s age ranged from 2 to 59 months.^31^
**Young infants.** young infants age ranged from 0 to 2 months.^31^
**Sick young infants.** These are infants aged 0 to 2 months with any signs and symptoms of illness who seek and/or receive medical care.^31^

### Data quality management.

The registers and chart booklets in which the case management procedures were recorded per the iCMNCI protocol and were used as quality assurance tools for assessment, classification, treatment, and follow-up. Data collectors were trained HEW supervisors and had previous experiences on how to use the PRCMM tools. Data collectors and peer-HEWs reviewed the registers against the guidelines, and one-to-one feedback and experience sharing sessions were held in a good spirit. The overall data collection was coordinated and supervised by trained program officers and supervisors.

### Statistical analysis.

Data were cleaned and edited manually before entered to MS Excel 2016 for descriptive analyses. Categorical variables were summarized in frequency tables and as percentages. The results were presented in tables and charts. The proportion of patients correctly classified, treated, and followed up were computed separately for infants aged 0 to 2 months and children aged 2 to 59 months.

### Ethical consideration.

Ethical clearance was obtained from the ethical review committee of Benishangul Gumuz Regional Health Bureau. Support letter was obtained from district health offices and health posts. To ensure anonymity of cases, names of patients were avoided during reviews of the registration book.

## RESULTS

### Study participants.

A total of 32,981 under-5 children received clinical care at 236 health posts from April to December 2019. A majority of the children (31,830; 96.5%) were aged 2 to 59 months, and 1,151 (3.5%) were young infants aged 0 to 2 months. More than half 17,430 (52.8%) were male. Of the 1,151 young infants, 227 (19.7%) were SYIs who presented with an illness, and 924 (80.3%) had no disease classification.

### Disease classification.

A majority of the children aged 2 to 59 months (28,164; 88.5%) presented with an illness, of whom 20,444 (72.6%) were classified as having pneumonia, diarrhea, and/or malaria. More than one-third 8,812 (31.3%) had diarrhea, with the majority 8,184 (92.8%) having no sign of dehydration. In addition, a quarter 5,112 (25%) of the children were classified as having pneumonia, of whom 31 (0.6%) had severe pneumonia. Little more than half 10,272 (50.2%) of the children aged 2 to 59 months were assessed for malaria of whom 6,520 (63.5%) were classified as having malaria as confirmed positive by a rapid diagnostic test (Table 1).

**Table 1:** Disease classification of young infants and children who received clinical care at the health posts, Benishangul Gumuz Region, Ethiopia, April–December 2019 (*N* = 32,981)

Disease classification	Sex		Total
	Male	Female	
	*n* (%)	*n* (%)	*n* (%)
Total consultations	17,430 (52.8%)	15,551 (47.2%)	32,981 (100%)
Total 2–59 months old children who received healthcare	16,824 (52.9%)	15,006 (47.1%)	31,830 (96.5%)
Total 0–2 months young infants who received healthcare	606 (52.6%)	545 (47.4%)	1,151 (3.5%)
Disease status of children aged 2–59 months			
No	2,179 (59.4%)	1,487 (40.6%)	3,666 (11.5%)
Yes	14,645 (52%)	13,519 (48%)	28,164 (88.5%)
Disease status of young infants aged 0–2 months (*N* = 1,151)			
No	486 (42.2%)	438 (38.1%)	924 (80.3%)
Yes	120 (10.4%)	107 (9.3%)	227 (19.7%)
Disease classification among children aged 2–59 months (*N* = 28,164)			
Pneumonia	2,658 (52.0%)	2,454 (48.0%)	5,112 (18.2%)
No pneumonia/cold cases	1,802 (52.0%)	1,663 (48.0%)	3,465 (12.3%)
Diarrheal diseases*	4,582 (52.0%)	4,230 (48.0%)	8,812 (31.3%)
Persistent diarrhea/very severe dehydration/dysentery	162 (51.8%)	151 (48.2%)	313 (1.1%)
Diarrhea with some dehydration	164 (52.1%)	151 (47.9%)	315 (1.1%)
Diarrhea with no dehydration	4,256 (52%)	3,928 (48.0%)	8,184 (29.1%)
Very severe febrile disease/illness	200 (52.1%)	184 (47.9%)	384 (1.4%)
Malaria†	5,341 (52.0%)	4,931 (48.0%)	10,272 (36.5%)
Malaria cases (RDT positive for PF or PV)	3,390 (52.0%)	3,130 (52.0%)	6,520 (23.2%)
Malaria cases (RDT not confirmed)	1,951 (52.0%)	1,801 (48.0%)	3,752 (13.3%)
Malnutrition‡	62 (52.1%)	57 (47.9%)	119 (0.4%)
New sever complicated malnutrition	10 (50.0%)	10 (50.0%)	20 (0.1%)
Sever uncomplicated malnutrition	42 (52.5%)	38 (47.4%)	80 (0.3%)
Moderate acute malnutrition (MAM)	10 (52.6%)	9 (47.4%)	19 (0.1%)
Type of diseases for sick young infants (*n* = 227)			
Local bacterial infection	46 (52.9%)	41 (47.1%)	87 (38.3%)
Diarrheal disease	34 (53.1%)	30 (46.9%)	64 (28.2%)
Very severe disease	25 (52.1%)	23 (47.9%)	48 (21.1%)
Very preterm and/or very low birth weight	13 (54.2%)	11 (45.8%)	24 (10.6%)
Severe jaundice	2 (50.0%)	2 (50.0%)	4 (1.8%)

PF = *Plasmodium falcifarum*; PV = *Plasmodium vivax*; RDT = rapid diagnostic test.

* Total cases of diarrheal diseases.

† Total cases of malaria.

‡ Total cases of malnutrition.

Of the 1,151 young infants aged 0–2 months, 227 (19.7%) were classified as having an illness and 924 (80.3%) received postnatal care or well-baby services (Table 1). Among SYIs, 87 (38.3%), 64 (28.2%), 48 (21.2%), and 24 (10.6%) were classified as having had local bacterial infection, diarrheal disease, very severe disease, and very low birth weight, respectively. These four diagnoses accounted for 98.2% of the disease classification among young infants (Table 1).

### Patient management.

A total of 19,888 children and SYIs were treated for some medical condition. One-quarter 5,112 (25.7%) were treated with amoxicillin for pneumonia and 2,136 (10.7%) were treated with a similar drug for cough/common cold. Of the 8,812 diarrhea cases, 5,827 (66.1%) received both ORS and zinc per the treatment protocol. In addition, 6,386 (97.9%) of 6,520 sick children with confirmed malaria by rapid diagnostic test received Coartem and 132 (3.9%) received chloroquine (Table 2). Among the 227 SYIs, 196 (86.3%) were treated at the health post and 31 SYIs (13.7%) did not receive any treatment. Among the 48 children with very severe diseases, only 15 (31.3%) initiated treatment at the health post and 14 (29.2%) received antibiotics, of whom 10 (71.4%) completed the course of medication. A majority of SYIs (74; 85.1%) who developed bacterial infection were treated with amoxicillin (Table 2).

**Table 2:** Treatment of children aged 0 to 59 months at the health posts, April–December 2019, Benishangul Gumuz Region, Ethiopia

Variables	Sex			
	Male	Female	Total	Percent
Total 2–59 months cases treated (*N* = 28,164)				
Yes	10,289	9,053	19,792	70.3%
No	4,537	3,835	8,372	29.7%
Cases treated with amoxicillin for 5 days (*n* = 7,248)				
Pneumonia cases treated with amoxicillin	2,658	2,454	5,112	70.5%
Nonpneumonia/cough/cold cases treated with amoxicillin	1,111	1,025	2,136	29.5%
Malaria case treated with standard treatment (*n* = 6,520)				
*Plasmodium falciparum* cases treated with Coartem	3,201	3,065	6,386	97.9%
*Plasmodium vivax* cases treated with chloroquine	132	122	254	3.9%
Diarrhea cases treated with ORS and zinc (*N* = 8,812)				
Yes	3,030	2,797	5,827	66.1%
No	1,552	1,433	2,985	33.9%
Children referred to health centers or hospitals in accordance with guideline recommendation				
Yes	40	37	77	100%
No	0	0	0	0%
Total 0–2 months sick young infants treated (*n* = 227)				
Yes	103	93	196	86.3%
No	17	14	31	13.7%
Very severe disease classification (*n* = 196)				
Yes	25	23	48	24.5%
No	78	70	148	75.5%
Very severe disease started treatment at health post (*n* = 48)				
Yes	8	7	15	31.3%
No	17	16	33	68.8%
Sick young infant referred to higher institutions (*n* = 33)				
Yes	13	12	25	75.8%
No	4	4	8	24.2%
Very severe disease received antibiotics (*n* = 15)				
Yes	7	7	14	93.3%
No	1	0	1	6.7%
Very severe disease completed antibiotics (*n* = 14)				
Yes	5	5	10	71.4%
No	2	2	4	28.6%
Local bacterial infection treated with amoxicillin (*n* = 87)				
Yes	39	35	74	85.1%
No	7	6	13	14.9%
Cases treated with ORS and zinc (*n* = 64)				
Yes	31	27	58	90.6%
No	3	3	6	9.4%
Others treatment not specified (consultation)*	17	14	31	

ORS = oral rehydration salt.

* Postnatal services provided.

### Compliance with the case management protocol.

#### Children aged 2 to 59 months.

**Figure 2.**
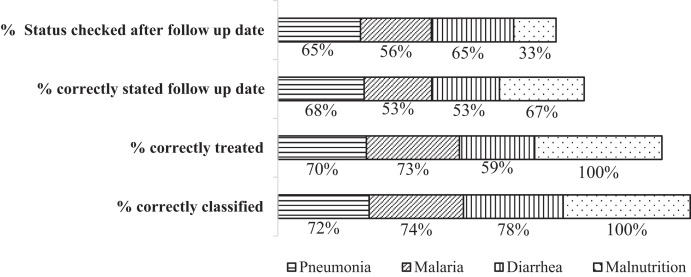
Compliance of child case management with Integrated Community Case Management of Newborn and Child Illness guideline recommendations among children aged 2 to 59 months in Benishangul Gumuz region, Ethiopia, April–December 2019 (*N* = 1,217).

The medical records of 1,217 cases were reviewed to assess whether the case management practices were per the treatment protocol. Accordingly, the case management of pneumonia, malaria, and diarrheal diseases showed deviation from the guideline recommendation. Of the 426 pneumonia cases reviewed, 306 (72%) were assessed and correctly classified as having had pneumonia, and 297 (70%) were treated with amoxicillin per the protocol. The follow-up dates of 289 (68%) pneumonia cases were stated and the status of treatment outcome was documented for 277 (65%) cases (Figure 2).

Among the 287 malaria cases, 213 (74%) were correctly classified, 210 (73%) were correctly treated, and 153 (53%) had documented follow-up dates per the treatment protocol. Of the 501 cases of diarrheal diseases, only 393 (78%) were correctly classified, 297 (59%) were correctly treated, and 267 (53%) cases had documented follow-up dates per the treatment guideline (Figure 2).

#### Infants aged 0 to 2 months.

**Figure 3.**
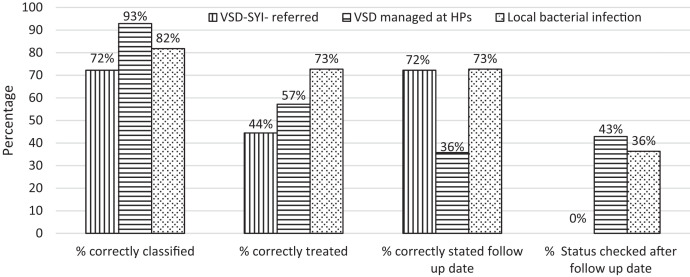
Compliance of sick young infant case management with the Integrated Community Case Management of Newborn and Child Illness protocol in Benishangul Gumuz region, Ethiopia, April–December 2019 (*N* = 43).

Among the 236 health posts included in the study, only 32 (13.6%) had seen one or more SYI case in which the medical records of 43 SYIs were reviewed to assess if the clinical management complied with the iCMNCI protocol (Figure 3). From 18 very severe disease cases, 13 (72%) were correctly classified as having very severe disease, and 8 (44%) SYI were referred to the next higher level for further investigation after they had received amoxicillin and gentamycin. Among SYIs managed at the health post, data of 14 young infants with danger signs and symptoms were reviewed; 13 (92%) SYI were correctly classified, eight (57%) were correctly treated with amoxicillin for 2 days and with gentamicin for 7 days, five (36%) were correctly provided with a follow-up date, and the outcome status was checked for six (43%) cases per the protocol (Figure 3).

Overall, compliance with guideline recommendations in the management of local bacterial infections declined from assessment through documentation of the follow-up date. Of the total 11 SYIs who had symptoms and signs of local bacterial infections, nine (82%) were correctly classified, eight (73%) were treated with amoxicillin for 5 days, and the follow-up date was documented for four (36%) cases (Figure 3).

## DISCUSSION

This study was conducted to assess the compliance of child health services with guideline recommendations in primary care settings where the iCMNCI guideline is implemented. Data abstracted from the iCMNCI registers showed that three diseases including pneumonia, diarrhea, and malaria accounted for 73% of the total diagnoses among children aged 2 to 59 months. This is in line with previous reports that the major burden of diseases among this age group are either preventable or treatable.^32^ Among the three major disease classifications, that is, pneumonia, malaria, and diarrhea, the treatment of some cases was not in accordance with guideline recommendations. Amoxicillin was inappropriately used for the treatment of cough or common cold in 2,136 (29.5%) children aged 2 to 59 months. During PRCMM sessions, HEWs said that the excessive use of amoxicillin in nonpneumonia cases was mainly due to parental influence to treat their sick children with antibiotics. Such findings are in alignment with results from a previously conducted study in Nairobi, Kenya.^33^ Misclassification of nonpneumonia cases (such as cough or common cold) as pneumonia could be another reason for the inappropriate use of amoxicillin.

Substandard treatment of diarrheal diseases was also common. According to the iCMNCI case management protocol, all children who have diarrhea with no, some, or severe dehydration should be treated with ORS and zinc.^32^ However, only 5,827 (66.13%) of the 8,812 children with diarrheal diseases were treated per the protocol. The low treatment coverage might be due to lack of regular refills by HEWs from catchment health centers or district health offices.

According to the iCMNCI guidelines, all newborns should be registered and provided with well-baby services at the health posts. For infants who have any of the danger signs or symptoms, the HEW should assess, classify, treat, and schedule follow-up dates.^32^ Community health workers, such as HEWs, are also mandated to visit homes of sick children if mothers or caregivers do not come to the health facility on the scheduled appointment date.^32^ However, in the 236 health posts, there were very low number of young infants aged 0 to 2 months who received well-baby services. This could be due to lack of registering the home-to-home postnatal visits, often made by HEWs. The other explanation could be nonadherence to the registration and chart booklet protocols by HEWs as evidenced during follow-up visits by supervisors from primary healthcare units or district health offices.

The proportion of children correctly assessed for pneumonia, malaria, and diarrhea were 72%, 74%, and 78%, respectively. Similarly, the proportion of cases correctly treated for pneumonia, malaria, and diarrhea were 70%, 73%, and 59%, respectively. These results were lower compared with previous study results in Tigray, Oromia, and Southern Nations, Nationalities, and Peoples’ Region (SNNPR) of Ethiopia where the proportion of pneumonia, malaria, and diarrheal cases that were correctly assessed per the guideline were 88%, 93%, and 92%, respectively.^34^ In addition, the proportion of cases correctly classified and treated for pneumonia, malaria, and diarrheal diseases were 86%, 91%, and 80%, respectively.^34^ This shows the continued challenges faced in the region as a result of weak health system. The other potential explanation is that health workers other than HEWs were assigned to provide clinical services without in-service orientation or training about the iCMNCI protocol. To improve consistency of the assessment, disease classification, treatment, and follow-up of cases, quality grid supportive supervision,^35^^–^^37^ training,^36^ and PRCMM are recommended.^35^^,^^36^ Such interventions have previously shown positive results in Jimma and West Hararghe Zones^35^ and in Amhara, Oromia, and SNNPR regions of Ethiopia.^35^^,^^36^

Documentation of follow-up dates and child health outcomes were assessed to have an insight on childhood improvements after treatment of major illnesses. The assessment findings showed that follow-up dates were correctly stated, and health outcomes were correctly checked in 43% to 65% of the diagnoses. This finding was relatively higher than a previous study conducted in Benishangul Gumuz Region where follow-up dates were correctly stated only in 12.6% of the cases.^37^ This needs to be strengthened because it may motivate families or caregivers to know their children’s health status after each episode of treatment.

This study has strengths. It assessed the compliance of iCMNCI services against guideline recommendations in rural primary care settings that covered 236 health posts. In addition, patient record reviews were conducted by supervisors, HEWs, and coordinators. This has helped to validate the results onsite.

The study also has limitations. The results from records review were not validated through reexamination of cases or direct observation of HEWS’ clinical practices. Some major diseases such as measles and jaundice were not included because such cases were either rejected during quality grid review or were absent. Outcomes of the follow-up were not recorded nor assessed for a substantial proportion of cases. Finally, data were obtained only from register review, and the behavioral factors of health care providers and healthcare utilizers were not assessed to complement and triangulate the quantitative description.

In conclusion, this study showed inconsistencies between iCMNCI recommendations and actual practices of sick child assessment, disease classification, treatment, and documentation of a scheduled follow-up visit date by HEWs. Follow-up visits were few, and none of the health posts had recorded child health outcomes during the follow-up visit. Therefore, it is recommended for front-line community health workers (such as HEWs) to strictly use the chart booklet during child assessment, disease classification, treatment, and follow-up and to comply with the iCMNCI recommendations. Health managers should focus on the capacity building of frontline community health workers to improve their knowledge, skills, and practices. In addition, HEWs should be supported to follow the iCMNCI case management protocol strictly and to use the chart booklet during assessment, classification, treatment, and follow-up of under-5 children through training, mentorship, and supportive supervision.
